# Behavioral facilitation and increased brain responses from a high interference working memory context

**DOI:** 10.1038/s41598-018-33616-3

**Published:** 2018-10-17

**Authors:** George Samrani, Petter Marklund, Lisa Engström, Daniel Broman, Jonas Persson

**Affiliations:** 1 Aging Research Center (ARC), Karolinska Institute and Stockholm University, Tomtebodavägen 18A, 171 65 Solna, Sweden; 2 Department of Psychology, Stockholm University, 106 91 Stockholm, Sweden; 3 School of Bioscience, University of Skövde, Högskolevägen, Box 408, 541 28 Skövde, Sweden; 4 Department of Educational Sciences, School of Education, Health and Social studies, Dalarna University, 791 88 Falun, Sweden

## Abstract

Many real-life situations require flexible behavior in changing environments. Evidence suggests that anticipation of conflict or task difficulty results in behavioral and neural allocation of task-relevant resources. Here we used a high- and low-interference version of an item-recognition task to examine the neurobehavioral underpinnings of context-sensitive adjustment in working memory (WM). We hypothesized that task environments that included high-interference trials would require participants to allocate neurocognitive resources to adjust to the more demanding task context. The results of two independent behavioral experiments showed enhanced WM performance in the high-interference context, which indicated that a high-interference context improves performance on non-interference trials. A third behavioral experiment showed that when WM load was increased, this effect was no longer significant. Neuroimaging results further showed greater engagement of inferior frontal gyrus, striatum, parietal cortex, hippocampus, and midbrain in participants performing the task in the high- than in the low-interference context. This effect could arise from an active or dormant mode of anticipation that seems to engage fronto-striatal and midbrain regions to flexibly adjust resources to task demands. Our results extend the model of conflict adaptation beyond trial-to-trial adjustments by showing that a high interference context affects both behavioral and biological aspects of cognition.

## Introduction

Even when we engage in an important task, our cognitive system may not always work at its peak. In reality, neuronal and behavioral responsiveness vary with changes in state and task difficulty^1–6^. A fundamental question is how cognitive and neural resources are coordinated and how executive control operations are flexibly adjusted to changes in task demands^7^.

The cognitive system needs to maintain current goal representations in WM while withstanding interference. Thus, the ability to select task-relevant information while inhibiting interfering or competing representations is crucial for successful task performance^8–10^. Adjustments of control after high-conflict trials have been explained within the framework of conflict monitoring, which proposes that experience of conflict can cause downstream upregulation of control processes^11–15^. In other words, conflict trials may induce interference control (IC), thereby affecting performance on subsequent trial(s). However the precise determinants of this adaptation to conflict on a trial-to-trial basis is unclear.

It has been hypothesized that internal control states are not primarily dependent on conflict as a driving force^16^. This alternative view suggests a more general and implicit level of learning that allows previous control states to be carried over to subsequent trials in a way that enhances performance. Thus, engagement of top-down control may not be a necessary prerequisite for subsequent performance enhancements. Instead, task-specific properties might provide a context to which an individual adapts by allocating necessary resources for performing the task as efficiently as possible^17,18^. Thus, conflict may be a task-specific property that gives rise to interference control and improves performance. Therefore, in addition to expected trial-to-trial adjustments, we aim to observe implicit adjustments as an affect that occurs across a whole task.

The conflict-monitoring hypothesis^11^ states that conflict adaptation reflects a feedback process whereby previous trial conflict is registered in the anterior cingulate cortex (ACC). Goal representation is then updated in a process subserved by the lateral prefrontal cortex (LPFC), leading to reduced interference on following trials. An alternative view is that the ACC is involved in a more general system that acts in response to cues signaling current and upcoming processing difficulty. Although transient effects have been attributed to activation in these regions^15,19–23^, the general effects of task-specific contexts (i.e. context enhancement) has received less attention. Adaptation of cognitive investment to task difficulty can also be achieved by a self-adjusting mechanism related to engagement of the anterior insula and inferior frontal gyrus together with the dorsal striatum^4^. Indeed, many brain regions involved in coping with increased task difficulty, overlap with those involved in executive and attentional control^24^. Basic mechanisms and strategic top-down processes, such as interference control, could both lead to enhanced information processing, and may work in parallel to optimize performance in highly demanding cognitive tasks.

In particular, little is known about the specificity of conflict adaptation. It has been proposed that the presence of conflict trials enhances domain-general task effort or attention, leading to better performance. Indeed, conflict adaptation effects sometimes generalize across contexts^25–29^. A recent study could observe improved encoding into memory for trials preceded by incongruent, or high conflict trials^23^. On the other hand, it has been argued that there are strict boundary conditions for when transfer of conflict adaptation occurs, and it is likely that transfer to alternate tasks are contingent upon shared across-task stimulus features and the overlap of task-information^30^. The extent to which control-demanding task-contexts also exert a general influence on trials with low-control demands is currently unknown. Can a high interference context facilitate an overall increase in performance on unrelated trial types within the same task? Most studies primarily quantify conflict adaptation as the difference in behavior on the current incongruent trial and the immediately preceding trial (n-1; either congruent or incongruent), but the contrast between high- and low-control task contexts is relatively unexplored.

In the present study, we used the recent-probes item-recognition WM task^31–35^ to test for context-sensitive effects on behavioral and neural outcome measures. It was important to use a single task in which interference can be varied along the same dimension, so that task demand can be manipulated independent of stimulus features. The principal aim was to uncover how such differences in task demand (presence or absence of high-interference trials) would affect behavioral performance and neural indicators associated with non-interference trials.

Attentional control states may have a long duration and transfer to other task contexts^36–38^, imposing a methodological problem in within-individual designs with intermixed high- and low-demand conditions. Experiments 1, 3, and 4 therefore used between-individual designs. In addition, experiment 2 used a within-individual design in which participants were tested in high- and low-demanding contexts on separate days.

Thus, in the first experiment, we employed a between-group design. Participants were randomly assigned to one of two groups: one performed a task that included high interference trials, and the other performed a task that did not. A second experiment aimed to replicate the effects of experiment 1 using a within-group design. In a third experiment, we increased the number of items to be maintained in WM from four to six to test whether the effect was also present when WM load was comparatively high. In a fourth experiment, we combined behavioral and functional magnetic resonance imaging (fMRI) measurements.

In particularly, we aimed to test (i) whether greater control demand caused by proactive interference (PI) has a general and perhaps sustained carry-over effect on all non-recent trials in a task, thus increasing performance, (ii) whether and to what extent control can be engaged implicitly, and (iii) whether greater activation in task-relevant brain regions is associated with performing the task in a high-demanding context. We hypothesized that PI results in increased performance on non-recent trials. On the basis of previous literature, we also predicted (i) that inclusion of high interference trials would increase activation in regions associated with conflict monitoring and cognitive effort, such as the anterior cingulate cortex (ACC)^11,39^ and (ii) that any performance facilitation observed in the behavioral experiments could be a result of increased general effort or motivation stemming from the more demanding high-interference condition. Several candidate regions have been implicated in translating incentives into cognitive motivation and effort, including dopamine regulating striatal regions, along with midbrain structures^39^. Finally, control regions involved in IC, such as the inferior frontal gyrus (IFG) may also play an integral part.

## Results of Experiments 1, 2, and 3

### Experiment 1

**Figure 1:**
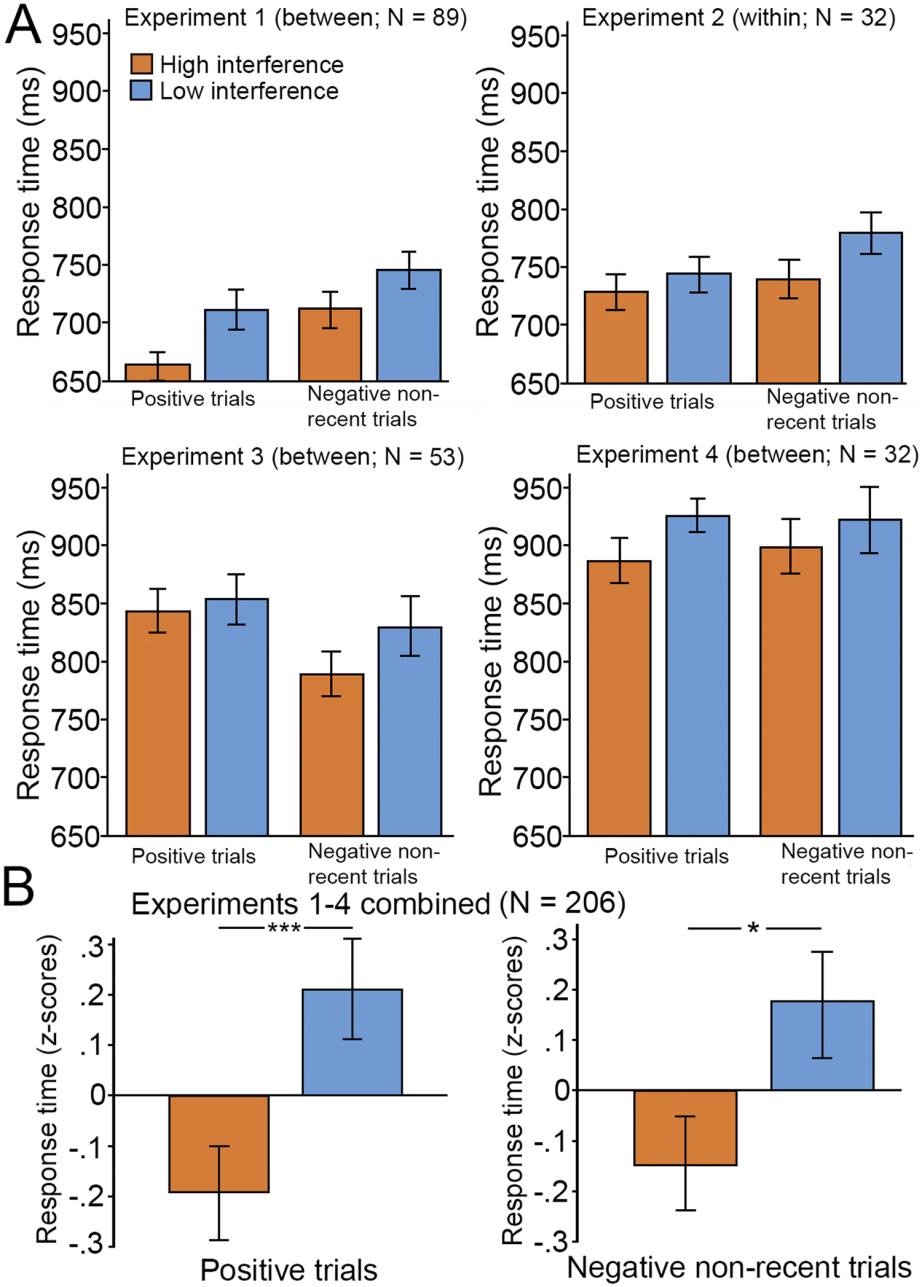
Task performance (reaction time) on non-recent probes (**A**) across the four experiments. (**B**) The combined normalized results (z-scores) of the four experiments show that participants who completed the high-interference (interference) version of the task performed better than those who completed the low-interference (control) version. Error bars indicate standard means across participants. Asterisks indicate significant main effects (*P < 0.05; ***P < 0.005).

First, a control analysis was performed in the high-interference group to ensure that this group was affected by interference from familiar probes. A repeated-measures ANOVA showed that reaction times (RTs) were reliably slower on recent negative trials than on non-recent negative trials (recent negative trials: 764 ms, non-recent negative trials: 711 ms; F(1,50) = 24.6, P < 0.001, η^2^ = 0.33). A 2 × 2 mixed ANOVA was conducted (Trial type [positive non-recent vs. negative non-recent] × Group [high vs. low interference]) to investigate the effect of contextual enhancement on RT for non-recent trials. The main effect of trial type (F(1,88) = 20.8, P < 0.001, η^2^ = 0.19) was significant, showing that RTs were faster for positive than for negative probes. In line with our hypothesis, there was a significant main effect of group (F(1,88) = 4.53, P = 0.036, η^2^ = 0.05), showing that participants in the high-interference group responded to non-recent probes more quickly than the low interference group (Fig. 1). The trial type by group interaction was not significant (F(1,88) = 1.31, P = 0.26, η^2^ = 0.02). Furthermore, planned comparisons showed that the difference between the high- and low interference groups was significant for positive (high interference: 654 ms, low interference: 711 ms; F(1,88) = 5.47, P = 0.022, η^2^ = 0.06) but not for non-recent negative probes (high interference: 711 ms, low interference: 745 ms; F(1,88) = 2.25, P = 0.137, η^2^ = 0.03).

**Figure 2:**
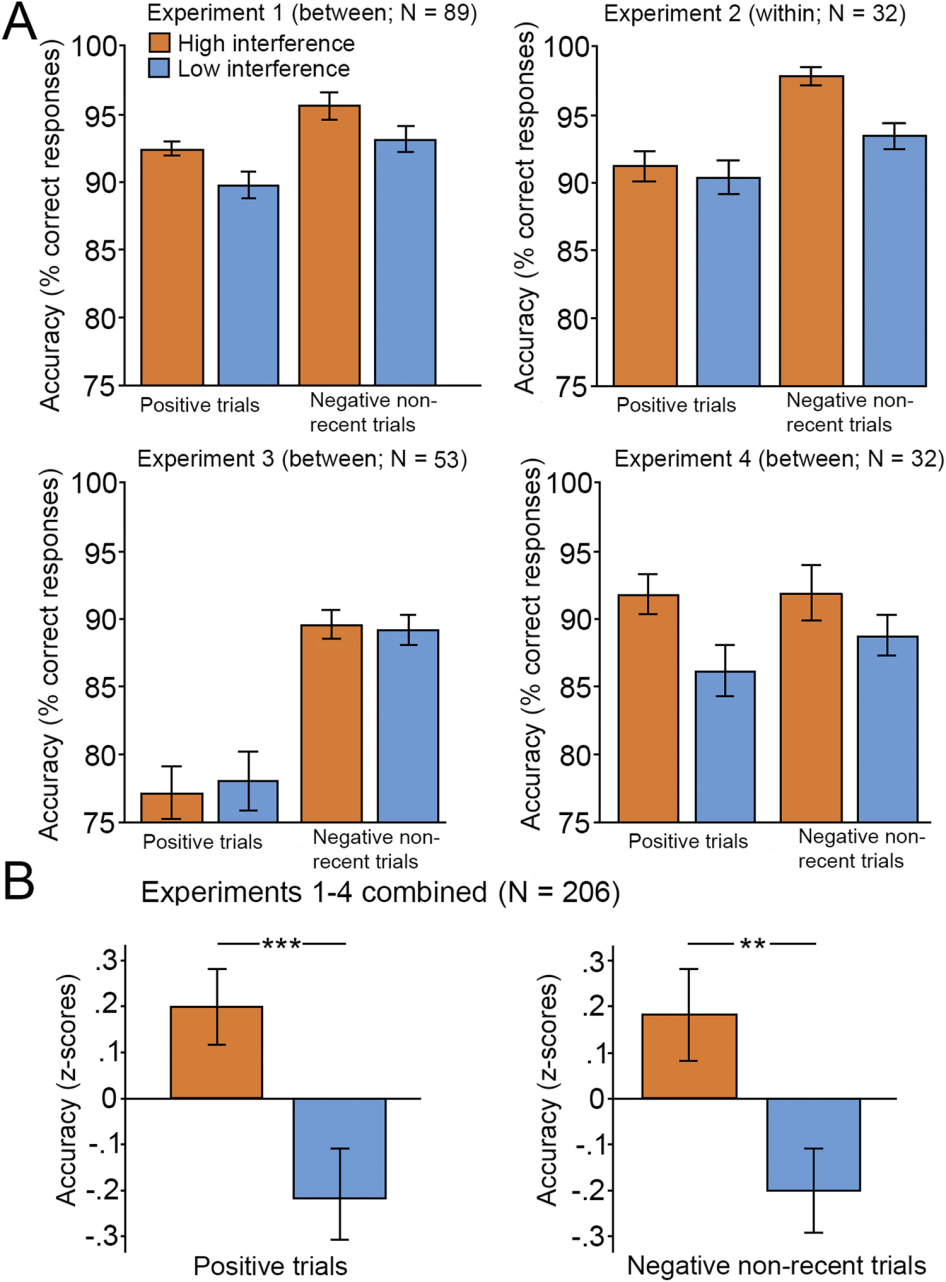
Task performance (accuracy) on non-recent probes (**A**) across the four experiments. (**B**) The combined normalized results (z-scores) of the four experiments show that participants who completed the high-interference (interference) version of the task performed better than those who completed the low-interference (control) version. Error bars indicate standard means across participants. Asterisks indicate significant main effects (**P < 0.01; ***P < 0.005).

The results of the analyses of accuracy were similar to those for RT. Accuracy was lower in interference than in non-interference trials (recent negative trials: 84.2%; non-recent negative trials: 95.8%, F(1,50) = 57.8, P < 0.001, η^2^ = 0.54). In addition, in a 2 (Trial type [positive vs. negative non-recent]) × 2 (Group [high vs. low interference]) mixed ANOVA, performance was higher for negative non-recent probes than for positive probes (F(1,88) = 19.9, P < 0.001, η^2^ = 0.03). Furthermore, the main effect of group was significant (F(1,88) = 6.62, P = 0.012, η^2^ = 0.07) and showed that the high interference group performed better than the low interference group (Fig. 2). The trial type by group interaction (F(1,88) = 0.006, P = 0.94, η^2^ = 0.00) was not significant. Planned comparisons revealed that the group difference was significant for positive probes (high interference: 92.5%, low interference: 89.8%; F(1,88) = 7.11, P = 0.009, η^2^ = 0.08), but the effect of negative non-recent probes was not significant (high interference: 95.8%, low interference: 93.2%; F(1,88) = 2.85, P = 0.095, η^2^ = 0.03). In summary, the analyses of response times and accuracy show that the inclusion of more difficult (high interference) trials improved WM and shortened RTs on non-recent trials.

Also, given observations of trial-to-trial adjustments in cognition for trials following high conflict trials, compared to low conflict trials e.g.^12,15,40^, we investigated whether reduced response times in the high-interference group was specific to non-recent probe trials following recent probe trials. Such adaptation effects in executive control could suggest that detection of conflict induce enhanced target processing on subsequent trials. However, in line with previous observations we found that response times were slower for non-recent negative trials that followed high interference (recent probes) trials compared to trials that followed low interference (non-recent negative or positive) trials (t(50) = 2.29, P < 0.05). A similar, but non-significant, effect was found for positive trials (t(50) = 1.61, P = 0.113). That is, no-interference trials following high-interference trials takes more time and are more difficult to answer than no-interference trials following no-interference trials. Therefore, the present results show context sensitive adjustments above and beyond negative trial-to-trial effect.

### Experiment 2

Similar to experiment 1, we observed that participants were slower on recent negative than on non-recent negative trials (recent negative trials: 727 ms, non-recent negative trials: 777 ms; F(1,31) = 31.3, P < 0.001; η^2^ = 0.50). A 2 × 2 mixed ANOVA (Condition [high vs. low interference] × Trial type [positive trials vs. negative non-recent trials]) was conducted to investigate the effect of context enhancement on RT on non-recent trials. There was a significant effect of condition (F(1,31) = 7.58, P = 0.010, η^2^ = 0.2), which showed that responses were faster for participants in the high- than in the low-interference condition. A significant main effect of trial type was found (F(1,31) = 5.53, P = 0.025, η^2^ = 0.15), showing that RTs were faster for positive probes than negative non-recent probes. In addition, a significant interaction effect was observed (F(1,31) = 5.08, P = 0.031, η^2^ = 0.14). That is, there was a larger difference between RT in high- and low-interference conditions for negative non-recent probes than for positive probes (Fig. 1). Planned comparisons showed that there was a difference between high- and low-interference conditions for non-recent negative (high interference: 738 ms, low interference: 776 ms; F(1,31) = 12.3, P < 0.001, η^2^ = 0.28), but not for positive probes (high interference: 727 ms, low interference: 741 ms; F(1,31) = 1.74, P = 0.19, η^2^ = 0.05).

Participants were less accurate on negative recent probes than on negative non-recent probes (recent probes: 93.7%, non-recent probes: 97.7%, F(1,31) = 13.4, P < 0.001, η^2^ = 0.3). In addition, A 2 (Trial type [positive vs. negative non-recent]) × 2 (Condition [high vs. low interference]) mixed ANOVA on accuracy showed a main effect for trial type (F(1,31) = 25.3, P < 0.001, η^2^ = 0.45). Responses were more accurate for positive probes than for non-recent negative probes. In line with our hypothesis, there was a main effect for condition (F(1,31) = 16.85, P < 0.001, η^2^ = 0.35). Accuracy was higher in the high- than in the low-interference condition. In addition, a significant interaction was observed (F(1,31) = 6.70, P = 0.015, η^2^ = 0.18): there was a larger between-condition difference for negative non-recent probes than for positive non-recent probes. Planned comparisons found a significant difference between high- and low-interference conditions for non-recent negative (high interference: 98%, low interference: 90%; F(1,31) = 17.39, P < 0.001, η^2^ = 0.36) but not for positive probes (high interference: 91%, low interference: 90%; F(1,31) = 1.05, P = 0.31, η^2^ = 0.03). Thus, we were able to replicate the findings of experiment 1 using a within-subject design.

### Experiment 3

A repeated-measures ANOVA showed that RTs were reliably slower on recent negative trials than on non-recent negative trials (recent negative trials: 848 ms, non-recent negative trials: 797 ms; F(1,26) = 31.9, P < 0.001, η^2^ = 0.55). As in experiments 1 and 2, we investigated the effect of high vs. low interference versions of the task for positive and negative non-recent probes. A 2 (Trial type [positive vs. negative non-recent]) × 2 (Group [high vs. low interference]) mixed ANOVA using RTs as the outcome measure found a main effect of trial type (F(1,51) = 16.10, P < 0.01, η^2^ = 0.24), showing that RTs for positive probes were slower than for negative non-recent probes. The main effect of group was not significant (F(1,51) = 0.82, P = 0.37, η^2^ = 0.02) indicating that the high- and low-interference groups performed similarly. The group by trial type interaction was not significant (F(1, 51) = 2.65, P = 0.11, η^2^ = 0.05). Planned comparisons of the difference between high- and low-interference groups found no difference between negative non-recent (Fig. 1; high interference: 788 ms, low interference: 830 ms; F(1,51) = 1.73, P = 0.20, η^2^ = 0.03) and positive probes (Fig. 1; high interference: 844 ms, low interference: 853 ms; F(1,51) = 0.11, P = 0.74, η^2^ < 0.01). The reaction times to negative non-recent probes were an average of 42 ms faster for the high- than the low-interference group (high: 830 ms; low: 788 ms), which is consistent with the results of experiments 1, 2, and 4 (Fig. 1).

Accuracy was investigated with a 2 (Trial type [positive vs. negative non-recent]) × 2 (Group [high vs. low interference]) mixed ANOVA. The analysis showed a general main effect of trial type; accuracy was higher for non-recent negative probes than for positive probes (F(1,51) = 88.90, P < 0.01, η^2^ = 0.64). However, there was no difference between the two groups (F(1,51) = 0.02, P = 0.90, η^2^ = 0.01) in terms of overall accuracy, which shows that regardless of group, participants performed similarly in both types of trials. Further, the trial type by group interaction was not significant, F(1,51) = 0.26, P = 0.61, η^2^ = 0.01. Planned comparisons of the high- and low-interference groups showed no difference between the negative non-recent (Fig. 2; high interference: 90%, low interference: 89%; F(1,51) = 0.05, P = 0.83, η^2^ < 0.01) and positive probes (Fig. 2; high interference: 77%, low interference 78%; F(1,51) = 0.1, P = 0.78, η^2^ = 0.01).

Experiment 3 showed that participants took longer to answer the positive than the non-recent negative probes and that their answers were less accurate. This difference was observed regardless of whether they were exposed to a low- or high-interference version of the 6-letter-load item recognition task. Thus, increasing WM load attenuated the effects observed in experiments 1 and 2.

## Results of Experiment 4

### Behavioral results

Analyses of RT confirmed the presence of PI in the high-interference group. A one-way repeated measures ANOVA showed that RTs on recent negative trials (median RT = 953.2) was significantly slower than RTs on non-recent negative trials (median RT = 884.4; F(1, 14) = 8.88, p = 0.01, η^2^ = 0.4). To investigate the effect of high- and low-interference versions of the task on RT in non-interference trials, a 2 (Trial type [positive vs. negative non-recent]) × 2 (Group [high vs. low interference]) mixed ANOVA was conducted. None of the main effects were significant (trial type (F(1,31) = 0.05, P = 0.82, η^2^ = 0.01), group (F(1,31) = 1.4, P = 0.25, η^2^ = 0.04)); the trial type by group interaction was also non-significant (F(1,31) = 0.24, P = 0.63, η^2^ = 0.01). However, data showed that the reaction times were an average of 30.5 ms faster in the high- than in the low-interference group (Fig. 1), which was consistent with results of studies 1 and 2. The lack of statistically reliable effects in the imaging experiment may be attributed to low power. Planned comparisons showed that the difference between the high- and low-interference groups was not significant for positive probes (high interference: 888 ms, low interference: 926 ms; F(1,31) = 2.68, P = 0.11, η^2^ = 0.08) or non-recent negative probes (high interference: 900 ms, low interference: 922 ms; F(1,31) = 0.36, P = 0.55, η^2^ = 0.012).

Analyses of accuracy confirmed that participants in the high-interference group performed less accurately on recent negative than on non-recent probes (recent probes: 78%, non-recent probes: 93%; F(1,15) = 23.8, P < 0.001, η^2^ = 0.63). In addition, a 2 (Trial type [positive vs. negative non-recent]) × 2 (Group [high vs. low interference]) mixed ANOVA with accuracy as the outcome measure did not show a main effect of trial type (F(1,31) = 1.01, P = 0.33, η^2^ = 0.03) or a trial type by group interaction effect (F(1,31) = 0.83, P = 0.37, η^2^ = 0.03). However, the main effect of group was significant (F(1,31) = 4.43, P = 0.044, η^2^ = 0.13): the high-interference group performed better than the low-interference group (Fig. 2). Planned comparisons showed that the difference between the high- and low-interference groups was significant for positive trials (high interference: 91.2%, low interference: 86.2%; F(1,31) = 5.47, P = 0.026, η^2^ = 0.15) but not for negative non-familiar trials (high interference: 91.9%, low interference: 88.8%; F(1,31) = 1.5, P = 0.23, η^2^ = 0.05).

### Brain imaging results

**Figure 3:**
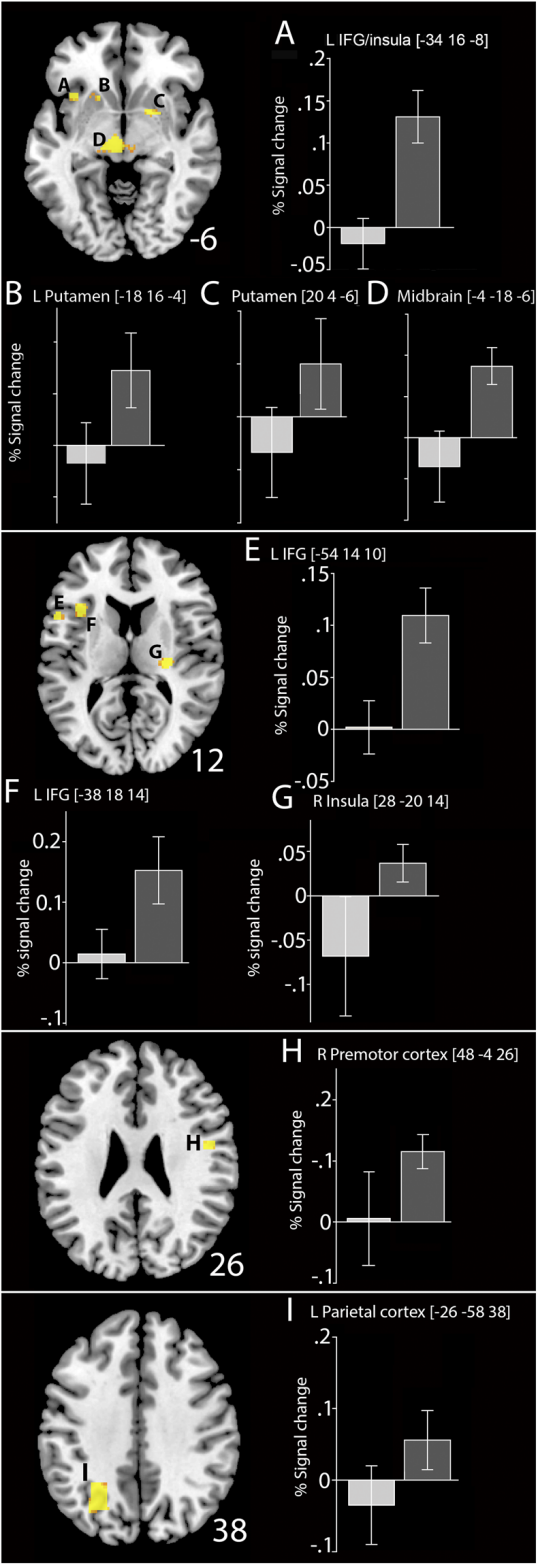
fMRI results. Differences in BOLD activity in individuals in the interference group (high-interference context; dark gray bars) and the control group (low-interference context; light gray bars). Between-group whole-brain results are based on first-level contrast of non-recent negative probes and positive probes (for display purposes, thresholded at P < 0.001 uncorrected). BOLD activation (% signal change) extracted from functional ROIs around the local cluster maxima. Error bars indicate standard means across participants.

We have reported the results of our analyses of brain activation related to interference (i.e. the difference between recent and non-recent negative probes in the high-interference group) elsewhere^34^. The current analysis compared differences in brain activation in high- and low-interference groups for non-recent probes. Separate analyses were performed for positive probes and non-recent negative probes. Given that the number of non-recent negative probes differed substantially between the groups (75% of the negative trials were recent probe trials in the high-interference group), we focused on the results of analyses of positive probes. A number of brain regions (Fig. 3; Tables 1 and 2) showed higher blood-oxygen level-dependent (BOLD) signal for positive probe trials in the high-interference group than in the low-interference group. These regions included the striatum (putamen), mesencephalic substantia nigra/ventral tegmental area (SN/VTA) of the midbrain, bilateral IFG, insula, premotor cortex, supplementary motor area/anterior cingulate cortex (SMA/ACC), angular gyrus, and medial temporal lobe (MTL). Additionally, we observed significantly greater deactivation in two regions of the default-mode network in the high- than the low-interference group: the caudal posterior cingulate cortex/precuneus and medial ventral PFC. Group differences (high interference > low interference) for non-recent negative probes included activations in the bilateral putamen, left thalamus, supplementary motor area, and insula.

**Table 1:** MNI coordinates for areas that show significant differences for positive probes between individuals in the high- and low-interference groups.

Anatomical localization	BA	x	y	z	mm^3^	*t*
**High interference group > low interference group**						
R Midbrain		4	−30	−12	3576	5.42
*L Midbrain (SN/VTA)*		*−6*	*−*16	*−4*		*5*.*18*
*L MTL/Hippocampus*		*−16*	*−26*	*−6*		*4*.*18*
L IFG	44/45	−38	18	14	2048	5.33
*L anterior insula*		*−34*	*16*	*−8*		*4*.*10*
R posterior insula		28	−20	14	856	5.25
*R thalamus*		20	−20	6		4.03
L Parietal cortex	40	−26	−58	38	2528	5.18
L IFG	44/45	−54	14	10	840	4.94
L SMA/ACC	32	−8	16	48	720	4.86
*L SMA*	6	−2	10	56		3.56
Cerebellum		0	−56	−32	1496	4.74
R Striatum (GP/putamen)		20	4	−6	456	4.36
R Precentral gyrus	6	48	−4	26	832	4.31
L Paracentral lobule	6	−16	−24	64	672	4.18
**Low interference group > high interference group**						
L posterior cingulate cortex/precuneus	31	−6	−66	18	520	3.74
Medial prefrontal cortex	10/32	0	38	4	712	3.68

x, y, and z are stereotactic coordinates. All reported regions survived an uncorrected threshold of P < 0.001 (FWE cluster corrected at P < 0.05). ACC = anterior cingulate cortex, BA = Brodmann’s area, GP = globus pallidus, IFG = inferior frontal gyrus, L = left, MTL = medial temporal lobe, R = right, SMA = supplementary motor area, SN = substantia nigra, VTA = ventral tegmental area.

**Table 2:** MNI coordinates for areas that show significant differences for non-recent negative probes between individuals in the high- and low-interference groups.

Anatomical localization	BA	x	y	z	mm^3^	*t*
**High interference group > low interference group**						
R Putamen		22	−2	−2	1326	4.60
R Supplementary motor area	6	12	−28	56	1168	4.19
*R Supplementary motor area*	6	*−4*	*−18*	54		*4*.*12*
R Putamen		24	20	2	576	4.10
L Putamen		−18	16	−4	464	4.01
L Thalamus		−16	−18	6	1416	3.80
L Insula/IFG	44/45	−36	16	10	1304	3.74
L IFG	44	−54	0	26	400	3.61
R Precentral gyrus	6	30	−10	38	432	3.54
L Middle occipital gyrus	7	−24	−58	40	624	3.38

x, y, and z are stereotactic coordinates. All reported regions survived an uncorrected threshold of P < 0.001 (FWE cluster corrected at P < 0.05). BA = Brodmann’s area, IFG = inferior frontal gyrus, L = left, R = right.

### Complementary analysis using behavioral data from all experiments

In a complementary analysis, we combined behavioral data from all four experiments to increase power and estimate overall context effects. Detailed methods for and results of these analyses can be found as supplementary information. Additional measures of accuracy were calculated as a discriminability index (*d*’), which represent the proportion of hit rates corrected for false positive rates^41^. Response bias was estimated using *c*^42^, which is defined as the distance between the response criterion and the neutral point, where neither response is preferred (value of 0).

We investigated the overall RT effect across experiments with a 2 (Group [high vs. low interference]) × 2 (Trial type [positive vs. negative non-recent]) × 4 (Experiment [experiments 1–4]) mixed ANOVA. The significant main effect of Group (F(1,198) = 7.16, P < 0.01, η^2^ = 0.04) indicated that RTs on non-recent trials were lower for participants in the high- than the low-interference group (Fig. 1). There was no main effect of Trial type (F(1,198) < 0.01, P = 0.95, η^2^ < 0.01); RTs for the two trial types (positive or negative non-recent) did not differ. The main effect of Experiment was not significant (F(3,198) = 0.02, P = 1, η^2^ < 0.01); RTs were similar across experiments. No interactions were significant.

Accuracy across experiments was tested using a 2 (Group [high vs. low interference]) × 2 (Trial type [positive vs. negative non-recent]) × 4 (Experiment [experiments 1–4]) mixed ANOVA. There was a significant main effect of Group (F(1,198) = 13.65, P < 0.001, η^2^ = 0.07), showing that performance was better in the high- than in the low-interference group (Fig. 2). There was no main effect of Experiment (F(3,198) = 0.29, P = 0.99, η^2^ < 0.01; accuracy was similar across experiments. The main effect of Trial type was not significant (F(1,198) < 0.01, P = 0.96, η^2^ < 0.01); that is, mean accuracy was similar for positive and negative non-recent trials. None of the other interaction effects were statistically significant (all Ps > 0.13).

To compare discrimination ability (*d*′) between the groups, we performed a repeated measures ANOVA with the factors Group [high vs. low interference] × Experiment [experiments 1–4]. There was a significant main effect of Group (F(1,198) = 4.3, P = 0.039, η^2^ = 0.02). Discrimination was higher for the group of participants that received the recent-probes task with interference trials than the low-interference group. Moreover, there was a main effect of Experiment (F(3,198) = 39.78, P < 0.001, η^2^ = 0.37); discrimination differed between experiments. The Group by Experiment interaction was not significant (F(3,198) = 1.55, P = 0.21, η^2^ = 0.02), showing that the groups did not differ significantly in discrimination between experiments. For response bias index *c*, the main effect of experiment was significant (F(3,198) = 38.26, P < 0.001, η^2^ = 0.38), but no other effects were significant (all Ps > 0.05). The significant group differences in *d*′ show that participants in the high interference context were better at discriminating targets from lures. A high-interference context did not affect whether participants adopted a conservative or liberal response bias.

## Discussion

This study provides behavioral and neural evidence that a high-interference context could serve as an implicit signal to allocate processing resources to the task, thereby improving WM performance. Using the recent-probes WM task, in which the level of PI was manipulated on a trial-by-trial basis, we showed that performing a high-interference version of the task increases performance on task-relevant non-interference trials. Behavioral facilitation resulting from a high-interference WM context was associated with higher brain activation in the ACC/SMA, IFG, striatum, SN/VTA, insula, MTL, and parietal cortex. These results suggest that when people adjust to increased interference and/or control demand, they engage the cognitive resources and neural circuitry important to maintaining high-level performance.

The current results are in general agreement with the conflict monitoring theory^11^. When people encounter familiar trials in which the need for IC is high, they devote more attention to processing subsequent target stimuli. Thus, greater interference in one trial may modulate performance by increasing the processing of subsequent stimulus sets. Our findings also suggest that the presence of conflict leads not only to trial-to-trial effects, but also to sustained processing that increases overall performance, perhaps by proactively engaging cognitive processes^43–45^. Important to note is that the enhanced performance seen in a high interference context was in the opposite direction of known trial-to-trial effects or conflict adaptation effects. Thus, the difference in performance between the high- and low-interference groups on non-recent trials was over and above the negative trial-to-trial effects on low interference trials that followed high interference trials. Instead, the behavioural consequence of contingency effects appeared to make participants’ responses transiently more conservative to ascertain that the probe was indeed a member of the current target set. Intriguingly, context-sensitive facilitation effect on non-recent trials was observed despite this effect. This indicates that increased performance was not specifically related to trials preceded by high interference trials. Rather, performance adjustments in the high interference group seem to rely on sustained engagement of cognitive control processes throughout the task.

Although the present study cannot disentangle the exact mechanisms underlying context adjustment, our finding point to increased attention to relevant stimulus information^20^. The present results suggest that enhanced task engagement is triggered implicitly rather than voluntarily, as participants were most likely not aware of the task manipulation (high- vs. low-interference trials). Several previous studies that did use the recent-probes task have demonstrated that participants are largely unaware of the conflict in the task^46,47^. Thus, this finding is in line with proposals that upregulation of processing resources can operate automatically and largely via implicit cues^48,49^. That is, we show evidence for a conflict-triggered attentional system that may increase maintenance of internal representations in WM, resulting in enhanced performance on other low conflict trials. Our results therefore extends earlier work showing that external incentives and rewards increase motivation and performance in participants who are aware of whether a particular task or condition is related to high or low incentives/rewards^50,51^. Our results also provide novel insights into current discussions on the interplay between representations in WM and visual attention to external stimuli^52^. That is, externally and internally directed attention appear to have similar properties and regulatory mechanisms. Many studies have found that experimentally manipulating variables such as effort^53–55^, task difficulty^5,56^, task preparation^57^, alertness^58^, and motivation^59^ systematically affects neurocognitive measures. Most commonly, such variables are considered non-selective and may be related to components of attention, such as the ability to achieve and maintain general readiness to respond to incoming information^60^. As a consequence, and somewhat paradoxically, increased demand for IC can result in faster response times and fewer errors on low conflict trials.

In the current study, on the neural level, the presence of interference resulted in increased activation in the IFG, striatum, parietal cortex, insula, and the mesencephalic SN/VTA region. These regions have consistently been linked to attentional control^61–66^. PFC activation has been implicated in maintaining task sets^67–71^ and may therefore play a part in regulating task-relevant processes. Initiating and maintaining a task set or preparing for a specific task is also associated with activation in PFC regions, and sustained frontal pre-task activation has been observed during WM performance^1^.

These results are particularly interesting in light of findings showing that anticipation of a WM update is related to activation of dopaminergic circuits that include the midbrain and putamen, as well as activation of the parietal cortex^3^. It could be that anticipating an update generates sustained activation in the midbrain and striatum that may relate to tonic dopamine up-regulation for the purpose of maintaining a relevant task set. Indeed, recent *in-vivo* observations in humans suggest that the dopaminergic midbrain, in conjunction with cortical control regions, is involved in flexible behavioral adjustments^72^ and resource recruitment^5,72^ in the absence of any external reward. Moreover, studies have demonstrated effort-related activation in the dopaminergic midbrain and putamen^73,74^. These regions overlap with those activated in the current study, further supporting the hypothesis that activation may be driven primarily by general mechanisms involved in task effort. BOLD activity in these areas has been previously correlated with dopamine levels using positron emission tomography^75^, and they are known as target regions for dopaminergic projections originating in SN/VTA^76^. The finding of greater activation in both the SN/VTA and striatum in the high-interference context supports the assumption that differences between the groups may be related to differential engagement of dopaminergic pathways. Therefore, the BOLD signal increases observed in our study may reflect increased firing of dopaminergic neurons in a high- compared to low-interference context.

A possible explanation underlying the current results is that high-interference contexts lead to increased associative binding of memory representations^77,78^ similar to Hebbian associative learning^79^. This view aligns with our proposal that interference trials lead to increased processing of target representations and contextual binding of these representations in WM to offset interference. Binding of context-item details is crucial to distinguishing familiar from unfamiliar probes. If resolving interference relies on keeping track of the temporal order of trials^9^, then associative binding may also be involved in encoding target information and thus improve performance on low-interference trials. Indeed, given that contextual binding has repeatedly been associated with activation of left IFG^80–84^ and the hippocampus^85–87^, our finding that these regions were activated in the high-interference group supports the hypothesis that a high-interference context leads to increased binding of active target representations in WM. Although most previous studies on contextual binding have involved episodic memory tasks, evidence suggests that these regions may also be involved in WM binding^88^. Moreover, studies suggest that binding of concrete stimulus features could also extend to include binding of current internal states; for example, attentional control settings^16,89^, and that such “event files” engage the hippocampus and putamen.

Some study limitations need to be considered when interpreting our findings. A limitation of the present study is the small sample size in experiment 4, and the results from this experiment should therefore be considered preliminary. Future studies with larger sample sizes are needed to confirm our brain imaging findings. Also, while we observed significant behavioral effects of contextual enhancement in experiment 1 and 2, these effects were much smaller in experiment 4, and absent in experiment 3. We believe that the lack of a statistically reliable behavioral effect in experiment 4 could be a result of low power in the fMRI experiment. In experiment 3, in which task difficulty was higher because of increased WM load, the effect of the interference trials was less pronounced. While this speaks against a *general* effect of context enhancement, we believe that there are boundary conditions for when this effect occurs. One interpretation could be that when task difficulty increases, there is less room for contextual enhancement. A similar argument has been put forward by Braver and colleagues^45^, who suggest that when WM load is low, people can use a proactive strategy for maintaining information in WM. When WM load is increased, they may shift to a reactive strategy that relies on familiarity-based recognition of the probe. Another possibility for the discrepant findings in experiment 3 is that a higher WM load itself results in increased within-task interference because of increased recycling of target letters. In line with this view, it has also been shown that additional time allowed for rehearsal of target items can result in more robust WM representations, leading to higher familiarity for subsequent lure trials. Higher familiarity might then be a cause to increased interference^13^.

Here we show that within-trial interference can be used as a cue to engage processing resources when responding to other trial types in the same task. This indicates that general control processing is context-specific, sensitive to task demands caused by interference from goal-irrelevant trials, and can operate in the absence of awareness. It further suggests that engagement of additional cognitive resources for target processing can arise not only as a result of extrinsic, but also of intrinsic motivating factors. We propose that a high-interference context leads to increased processing of target representations and contextual binding of these representations in WM. This study also provides fMRI evidence that fronto-striatal regions, along with the hippocampus and midbrain, play a key role in flexibly adjust resources to high-conflict demands. The present results add to our understanding of executive control processing and may offer novel approaches for optimizing human cognition.

## Material and Methods

Note that across all experiments, non-interference trials are referred to as “positive probes” (yes response) and “non-recent negative probes” (no response). In all experiments, participants were instructed to encode and maintain the target set into WM, and then indicate whether a single probe was part of the target set (Old) or not (New). They were instructed to answer as fast and accurately as possible. Across all experiments, median RTs were used to avoid excessive influence from deviant reaction times. Error trials and omissions were excluded from the RT analyses.

### Experiment 1

#### Participants

Eighty-nine young adults (20 men; age range: 18–30 years) were recruited from the Stockholm University community through posted advertisements. After they were given written and oral information about the study, all participants gave written informed consent. All procedures were performed in accordance with the relevant guidelines and regulations and approved by the Regional Ethical Review Board in Stockholm. They received course credits and/or payment for their participation. All participants were native Swedish speakers and had normal or corrected to normal vision. Participants were assigned to one of two groups: one received the high-interference version of the recent-probes task (N = 51), and the other group received a low-interference version of the recent-probes task (N = 38). The groups were matched for age and sex.

#### Procedure

Participants were tested individually in a dimly lit, sound-attenuated room. Stimuli were presented on a 15-inch monitor at a 50-cm viewing distance. E-prime software was used (Psychology Software Tools, Pittsburgh). A researcher described the task to the participant, who completed a short practice block before the actual test session started. In the recent-probes task, 144 trials were divided into three 48-trial blocks with a one-minute rest period between blocks. Each trial began with four lowercase letters and a central fixation cross presented in a square configuration for 1500 ms. A 1500-ms probe followed a 3000-ms delay and consisted of a single uppercase letter. On 50% of the trials, the probe was a member of the current target set, and on 50% of the trials it was not. Participants responded “yes” for a match with their right index finger, or “no” for a mismatch with their right middle finger. The inter-trial interval was 1500 ms, and there were no more than two consecutive positive or negative trials. In the low-interference condition, negative probes (6 per block) were neither in the current target set nor in the target set of the previous two trials. In the high-interference condition, the probes were either (i) a letter in the previous target set but not in the set before that (familiar/high interference, 12 per block) or (ii) a letter in the previous two trials (highly familiar/very high interference, 6 per block). Positive probes did not overlap with targets from the previous two trials and did not differ between the two groups. For all subsequent analyses, trials with both familiar and highly familiar probes were considered high-interference trials. The total time for the recent-probes tasks was approximately 18 min. Each participant’s median reaction time (RT) and number of accurate responses were used as outcome measures. Participants were not aware of the manipulation of conditions. After the test session, participants were allowed to ask questions about the tasks and were debriefed about the purpose of the experiment.

### Experiment 2

#### Participants

Thirty-two healthy young adults (16 men; age range 19–28 years) were recruited from the Skövde University community. After they were given written and oral information about the study, all participants provided written informed consent. All procedures were performed in accordance with the relevant guidelines and regulations and approved by the Regional Ethical Review Board in Stockholm. The participants received course credits and/or payment for taking part in the study. All participants were native Swedish speakers, right-handed, and reported normal or corrected to normal vision. In a randomized counterbalanced within-subjects design, participants performed a high- and a low-interference version of the recent-probes task. To avoid context effects that might occur in a test session, participants were tested on two separate days.

#### Procedure

This experiment was primarily carried out to replicate the findings of experiment 1 using a within-subjects design that would control for potential confounds related to baseline differences between the two groups. Participants were tested individually, by one and the same experimenter, and the experiment took place in a quiet and dimly lit room. Stimuli were presented on a 19-inch computer monitor with a viewing distance of 50 cm, using the E-prime software (Psychology Software Tools, Pittsburgh). The experimenter described the task to the participant, who completed a short practice block before the actual test session started. All participants performed the high- and low-interference version of the recent-probes task (described in experiment 1) in two separate test sessions, at the same time of day on two consecutive days. Each session was followed by a non-task related test, and the total testing time was approximately 40 minutes. Participants were randomly assigned to begin with either the high-interference version or the low-interference version of the item-recognition task. Participants were not aware of the manipulation of conditions. After the second session, participants were allowed to ask questions about the tasks and were debriefed about the purpose of the experiment.

### Experiment 3

#### Participants

Fifty-three young adults (17 men; age range: 19–30 years) were recruited from the Stockholm area in Sweden through posted advertisements and web pages. After they were given written and oral information about the study, all participants gave written informed consent. All procedures were performed in accordance with the relevant guidelines and regulations and approved by the Regional Ethical Review Board in Stockholm. Participants received course credits and/or payment for taking part in the study. All participants were native Swedish speakers and had normal or corrected to normal vision. Participants were randomly assigned to one of two groups: one group received the high-interference version of the recent-probes task (N = 26), and the other group received the low-interference version of the recent-probes task (N = 27). The groups were matched for age and sex.

#### Procedure

Because of the high accuracy and relatively fast response rates observed in experiments 1 and 2, we reasoned that the effects of the experiments could perhaps be driven by general task difficulty in addition to the inclusion of interference trials. Previous research suggests that when WM load is low, people can use a proactive strategy to maintain memory representations, but when load increases, they may shift to a reactive familiarity-based strategy instead^45^. A more demanding task could potentially reduce context effects by leaving less room for improvement when attentional or other control processes are highly allocated. We therefore expected that the influence of a high-interference WM context would be reduced if WM demand were high. In the third experiment, we therefore increased WM demand by using a WM load of 6 letters to examine whether results similar to those of experiments 1 and 2 could be obtained when cognitive demands were increased.

Participants were tested individually, by one and the same experimenter, and the experiment took place in a quiet and dimly lit room. Stimuli were presented on a 15.4-inch laptop computer screen (Compaq nx 7300) at a 50-cm viewing distance. E-prime (Psychology Software Tools, Pittsburgh) was used for stimulus presentation. Participants used their right index finger to respond “yes” to a match and their right middle finger to respond “no” to a mismatch. The experimenter described the task to the participant, who completed a short practice block before the actual test session started. Two-hundred trials were divided into five 40-trial blocks with an 8-second rest period between blocks. Each trial began with six lowercase consonant letters and a central fixation cross presented in a square configuration for 3000 ms, followed by a delay period of 3000 ms, a probe for 1500 ms, and an inter-trial interval of 700 ms. On 40% of the trials, the probe was a member of the current target set (positive probe), and on 60% of the trials it was not (negative probe). Participants responded “yes” for a match with their right index finger, or “no” for a mismatch with their right middle finger. Negative probes in the low-interference condition (12 per block) were neither in the current target set nor in the target set of the previous four trials. In the high-interference condition, the probes were either (i) a letter in the previous target set but not in the set before that (familiar/high interference, 6 per block) or (ii) a letter in the previous two trials (highly familiar/very high interference, 6 per block). Positive probes were all considered low-interference trials and did not overlap with targets from the previous two trials. For all subsequent analyses, trials with both familiar and highly familiar probes were considered high-interference trials. The total time for the task was approximately 27 min.

### Experiment 4

#### Participants

Thirty-two young adults (24 men; age range: 18–30 years) were recruited from the Umeå University community through posted advertisements. All participants were right-handed native Swedish speakers who reported no existing neurological or psychiatric illness. Vision was normal or corrected to near normal using MRI compatible glasses or contact lenses. After they were given written and oral information about the study, all participants gave written informed consent. All procedures were performed in accordance with the relevant guidelines and regulations and approved by the Regional Ethical Review Board in Stockholm. The participants received payment for their participation.

#### Procedure

Participants were randomly assigned to one of two groups: one performed a high-interference version of the recent-probes item-recognition task including negative recent probes, and the other group performed a low-interference version of the item-recognition task without recent probes. Participants first completed health screening over the telephone to ensure that it was suitable for them to participate in the study. fMRI scanning took place at the MRI research facility at Umeå University Hospital. Participants were given task instructions and completed a practice version of the task before the start of the scanning protocol. The groups were matched for age and sex. Additionally, a standardized intelligence test (Ravens Standard Progressive Matrices) was administered in a separate behavioral session. There were no group differences in performance on the intelligence test (t(29) = 0.31, P = 0.76).

#### fMRI tasks

Item recognition: The two groups performed different versions of the recent-probes item-recognition task (high vs. low interference). In contrast to the behavioral protocols used in experiments 1, 2, and 3, a variable length inter-trial interval (ITI) was used as follows: 72 ITIs of 1.5 sec, 36 ITIs of 3 sec, 18 ITIs of 4.5 sec, 12 ITIs of 6 sec, 3 ITIs of 7.5 sec, and 3 ITIs of 9 sec. Temporal jittering allowed for separation of independent trials in the fMRI analyses.

#### Image acquisition

The study was carried out using a Philips 3.0 Tesla high-speed echo-planar imaging device with a quadrature head-coil. For functional scanning, the following parameters were used: repetition time: 1512 ms (31 slices acquired), echo time: 30 ms, flip angle: 70°, field of view: 22 × 22 cm, matrix: 64 × 64, and slice thickness: 4.65 mm. To avoid signals arising from progressive saturation, ten dummy scans were performed prior to image acquisition. Structural high-resolution T1 images were also acquired: a 3D turbo field-echo sequence was used with the following parameters: repetition time: 10.5 ms, echo time: 5 ms, flip angle: 8 degrees, and field of view: 24 × 24 cm. One hundred and seventy sagittal slices with a slice thickness of 1 mm were acquired in 336 × 332 matrices. All images were sent to a PC and converted to Analyze 7.5 format.

#### Data analysis

Functional images were pre-processed and analyzed using SPM8 (Wellcome Department of Imaging Science, Functional Imaging Laboratory) implemented in Matlab 7.6 (Mathworks Inc., MA, US). After correcting for differences in slice timing within each image volume, all images were realigned to the first image volume acquired, normalized to standard anatomic space defined by the MNI atlas (SPM8), and spatially smoothed using a 8.0-mm full-width at half-maximum Gaussian filter kernel. Event-related effects were separately modelled in the framework of the general linear model (GLM) as implemented by SPM8. Event-related transient responses were modelled as regressors containing delta functions that represented onsets of the stimuli. All regressors of interest were convolved with the hemodynamic response function (HRF). Regressors of interest for the high interference group consisted of positive trials (n = 72), negative non-recent trials (n = 16), negative recent trials presented once (familiar; n = 32) and negative recent trials presented twice (highly familiar; n = 16). Regressors for the low interference group consisted of positive (n = 72) and negative (n = 72) trials. Covariates of no interest included the six realignment parameters, which accounted for motion artefacts. Single-subject statistical contrasts were set up using the GLM, and group data were analyzed in a random-effects model that differentiated between high-interference and low-interference groups. Statistical parametric maps were generated using t statistics to identify activated regions according to the model. Statistical parametric maps were also used to identify brain regions that exhibited reliable differences between the two groups receiving the high- and low-interference version of the recent-probes task. Groups were tested for significant differences using a family-wise error (FWE) cluster corrected threshold P < 0.05, based on an auxiliary uncorrected voxel-wise threshold of P < 0.001, resulting in a cluster threshold of 32 voxels (256 mm3). Cluster correction parameters were determined using SPM and a script (CorrClusTh.m; www2.warwick.ac.uk/fac/sci/ statistics/staff/academic-research/nichols/scripts/spm), which uses estimated smoothness (estimated FWHM 3.56 × 3.65 × 3.46 voxels = estimated FWHM 8 mm) and random field theory to find these corrected thresholds.

To further illustrate the fMRI results, we extracted the mean BOLD signal changes from selected regions of interest (ROIs) using the Marsbar toolbox (http://marsbar.sourceforge.net/). Functional ROIs were defined by including all suprathreshold voxels within a significant cluster derived from the SPM results at the group level. The parameter estimates were then used for plotting the results in SPSS, as well as for performing brain–behavior correlations. It should also be emphasized that the parameter estimates serve to illustrate group differences in regional activation patterns between individuals receiving the high-interference version of the task and those receiving the low-interference version, rather than being used for inferential statistics.

## Electronic supplementary material

Supplementary Information

## Data Availability

The datasets generated and analyzed during the current study are available from the corresponding author upon reasonable request.
