# Supplementary material for: Behavioral facilitation and increased brain responses from a high interference working memory context
Source: Sci Rep. 2018 Oct 17;8:15308. doi: 10.1038/s41598-018-33616-3 (PMC6193025; doi:10.1038/s41598-018-33616-3)
Supplement: Supplementary file 1 — Supplementary Information [file 41598_2018_33616_MOESM1_ESM.docx]

Behavioral facilitation and increased brain responses from a high interference working memory context.

George Samrani^1^, Petter Marklund^2^, Lisa Engström^3^, Daniel Broman^3,4^, and Jonas Persson^1,*^

^1^Aging Research Center (ARC), Karolinska Institute and Stockholm University, Tomtebodavägen 18 A, 171 65 Solna, Sweden; ^2^Department of Psychology, Stockholm University, 106 91 Stockholm, Sweden; ^3^School of Bioscience, University of Skövde, Högskolevägen, Box 408, 541 28 Skövde, Sweden; ^4^Department of Educational Sciences, School of Education, Health and Social studies, Dalarna University, 791 88 Falun.

**Supplementary information**

**Complementary analysis using behavioural data from all experiments**

Additional measures of accuracy was calculated as a discriminability index (*d*’), which represent the proportion of hit rates corrected for false positive rates[^1^](#_ENREF_1). Response bias was estimated using *c*[^2^](#_ENREF_2), which is defined as the distance between the response criterion and the neutral point, where neither response is preferential (value of 0). Since a within-subject design was used for experiment 2, we only included data from the first test session for each participant. Individual participant scores were converted to a normalized z-scores to facilitate comparability across tests. Negative values of *c* reveals a tendency to over respond (reflecting impulsivity/liberal response criteria) whereas positive values reveals a tendency to under-responding (cautious responding/conservative criteria). An advantage of using *c* is that it is unaffected by changes in *d*’ [^3^](#_ENREF_3). Because these measures are undefined when the proportion of responses equals 0 or 1, all responses were converted by adjusting the extreme rates. Rates of 0 are replaced with 0.5/*n*, and rates of 1 are replaced with (*n* - 0.5)/*n*, where *n* is the number of signal or noise trials[^4^](#_ENREF_4).

1 Snodgrass, J. G. & Corwin, J. Pragmatics of measuring recognition memory: applications to dementia and amnesia. *Journal of Experimental Psychology: General* **117**, 34-50 (1988).

2 Stanislaw, H. & Todorov, N. Calculation of signal detection theory measures. *Behavior Research Methods, Instruments, & Computers* **31**, 137-149 (1999).

3 Macmillan, N. A. in *A handbook for data analysis in the behavioral sciences: Methodological issues* (ed G. Keren & C. Lewis) 21-57 (Hillsdale, NJ: Erlbaum, 1993).

4 Macmillan, N. A. & Kaplan, H. L. Detection theory analysis of group data: estimating sensitivity from average hit and false-alarm rates. *Psychological Bulletin* **98**, 185-199 (1985).
